# DAssd-Net: A Lightweight Steel Surface Defect Detection Model Based on Multi-Branch Dilated Convolution Aggregation and Multi-Domain Perception Detection Head

**DOI:** 10.3390/s23125488

**Published:** 2023-06-10

**Authors:** Ji Wang, Peiquan Xu, Leijun Li, Feng Zhang

**Affiliations:** 1 School of Materials Science and Engineering, Shanghai University of Engineering Science, Shanghai 201620, China; ji_wang_sues@163.com (J.W.); zfshdd@163.com (F.Z.); 2 Shanghai Collaborative Innovation Center of Laser Advanced Manufacturing Technology, Shanghai University of Engineering Science, Shanghai 201620, China; 3 Department of Chemical and Materials Engineering, University of Alberta, Edmonton, AB T6G 1H9, Canada

**Keywords:** surface defect detection, object detection, dilated convolutional, attention mechanism, lightweight model

## Abstract

During steel production, various defects often appear on the surface of the steel, such as cracks, pores, scars, and inclusions. These defects may seriously decrease steel quality or performance, so how to timely and accurately detect defects has great technical significance. This paper proposes a lightweight model based on multi-branch dilated convolution aggregation and multi-domain perception detection head, DAssd-Net, for steel surface defect detection. First, a multi-branch Dilated Convolution Aggregation Module (DCAM) is proposed as a feature learning structure for the feature augmentation networks. Second, to better capture spatial (location) information and to suppress channel redundancy, we propose a Dilated Convolution and Channel Attention Fusion Module (DCM) and Dilated Convolution and Spatial Attention Fusion Module (DSM) as feature enhancement modules for the regression and classification tasks in the detection head. Third, through experiments and heat map visualization analysis, we have used DAssd-Net to improve the receptive field of the model while paying attention to the target spatial location and redundant channel feature suppression. DAssd-Net is shown to achieve 81.97% mAP accuracy on the NEU-DET dataset, while the model size is only 18.7 MB. Compared with the latest YOLOv8 model, the mAP increased by 4.69%, and the model size was reduced by 23.9 MB, which has the advantage of being lightweight.

## 1. Introduction

Steel accounts for more than 90% of all metals used in industrial production, because it is a material with high strength and ductility, and excellent manufacturability, at the lowest costs [1], ideal for machines, civil structures, transportation equipment, and endless list of tools [2]. As a metallic material [3], steel is widely used in manufacturing processes, such as brazing [4,5,6], laser welding [7,8], and additive manufacturing [9]. Surface defects in steel are often related to the microstructure changes during steel fabrication [10], and the interaction between alloying elements and microstructure can affect the formation of surface defects, thereby affecting the organization and mechanical properties of steels [11,12]. Steel surface defects may include cracks, bubbles, inclusions, scars, scratches. These defects will have a negative impact on the quality and performance of steel. These defects will reduce the strength, toughness, and ductility of the material, thereby affecting the service life and safety performance of the steel structures. Steel surface defects can also affect the appearance quality, product dimensions, and unstable performance [2]. By detecting and analyzing defects on the steel surface, potential safety hazards can be discovered in time, providing a basis for quality control and improvement in the production process. In addition, for different types of defects, corresponding measures can be taken to repair or deal with them, to improve the performance and reliability of steel products.

The traditional detection of steel surface defects is often carried out by manual visual inspection or physical inspection. However, visual inspection is easily limited by the resolution and fatigue of the human eye, making it difficult to find tiny defects, and the detection efficiency is low. However, physical inspection methods, such as liquid penetrant inspection (LPI) [13], magnetic particle inspection (MPI) [14], and ultrasonic inspection (UI) [15], are affected by inspection methods, steel materials with different physical properties, cost, and other factors, making it difficult to detect defects efficiently and quickly on steel surfaces.

Steel defect detection based on machine vision is a technology that applies computer vision technology and image processing algorithms to automatically detect and classify steel surface defects. This method usually needs to extract defect feature information from the image, such as color, texture, shape, and use edge detection [16], texture analysis [17], corner detection [18], and other technologies for feature extraction [19]. After the feature extraction, it is necessary to classify the features, and methods such as support vector machines [20], decision trees, naive Bayes, and clustering are commonly used for defect classification. However, practical applications have been limited in by factors such as image resolution, light source, and shooting angle.

In recent years, object detection algorithms have made great progress with the development of deep learning techniques. The target detection model based on the deep convolutional neural network has the ability of multi-level feature extraction and can learn more effective and rich feature expressions adaptively. For example, R-CNN [21] divides the image into multiple regions, and then classifies and regresses each region. Fast R-CNN [22] and Faster R-CNN [23] further improve the efficiency and accuracy of R-CNN. SSD [24] combines multiple feature layers to detect targets of different scales at the same time, improving the detection speed and accuracy. The YOLO series [25,26,27,28,29,30,31] predicts all objects in the image simultaneously through a single neural network, enabling real-time detection.

As lightweight models are widely proposed, such as SqueezeNet [32], MobileNet series [33,34,35], ShuffleNet series [36,37], EfficientNet series [38,39], ghostNet [40], MnasNet [41], and PeleeNet [42], the structure of these lightweight models is often used as the backbone structure of the target detection network. In the feature fusion structure, structures such as Feature Pyramid Network (FPN) [43], Path Aggregation Network (PAN) [44], and Bidirectional Feature Pyramid Network (BiFPN) [45] are common. The head network in target detection usually refers to the network module used for target detection output, which is used to convert feature maps into detection results, including the position, category, and other attributes of the target. For example, the Decoupled Head structure is used in YOLOX [30]. However, these models are not lightweight enough for industrial production. They are still too costly; they must use hardware with large computing resources; they cannot always perform real-time detection; and some do not have an efficient network structure.

Traditional convolutional neural network (CNN) usually uses a fixed convolution kernel on the input data to extract feature representations. Yet, traditional CNN has certain limitations, as it cannot effectively distinguish different objects or contextual information, resulting in limited accuracy in discrimination of feature representation. The common attention mechanisms are spatial attention [46] and channel attention [47,48,49]. Spatial attention can perform weighted fusion or enhancement of features according to different spatial positions, which are spatially aware. The dilated convolution [50] can increase the range of the receptive field to capture more global information without increasing the parameters of the convolution kernel. In the object detection task, the expansion of the receptive field is beneficial to the detection of large objects. Compared with traditional convolution, dilated convolution should be selected for locating the target boundary more accurately. It can also be shown to capture higher-order features of the input, which is conducive to the network’s understanding of semantic information and can improve the position accuracy of the final detection result.

Based on the above review, this paper addresses the technology gap by proposing an innovative model, DAssd-Net, for steel surface defect detection. The model is shown to fully consider the size of the receptive field, as well as the spatial and channel information of the feature map. Compared with other mainstream target detection models, the DAssd-Net can achieve 81.97% mAP accuracy on the NEU-DET dataset, while the model size is only 18.7 MB. The mAP index is 4.69% higher than the latest YOLOv8 model, while the model size is 23.9 MB smaller, and has more accuracy.

## 2. Related Works

### 2.1. Image Processing-Based Detection Method

Using traditional image processing methods to deal with steel surface defects usually requires the manual selection of parameters (such as threshold) and algorithms [51], and it is difficult to automatically adapt to the characteristics and needs of different images. According to the characteristics of steel surface defects, some scholars [52,53] designed or improved classic operators for detection accuracy. However, these methods cannot deal with the noise and distortions existing in the images, resulting in the degradation of the image quality after processing. To extract the features of steel surface defects more accurately, some studies [54,55] have designed more complex feature-extractors by combining multiple methods. These feature-extractors can improve the extraction of defect features and provide useful assistance for subsequent detection. However, these methods usually require many calculations and operations, the processing speed is slow, and it is difficult to detect in real-time.

### 2.2. Deep Learning-Based Detection Method

Traditional image processing methods require the manual design of feature-extractors, while deep learning methods can automatically learn features from data, thereby avoiding manually designing the feature-extractors [56,57]. More deep learning-based defect detection methods have been applied to steel materials. In actual steel surface defect detection, a diversity of defect sizes and shapes, object backgrounds, and complex lighting environments are encountered.

For small-sized defects, the surface texture and color changes are also relatively small, so it is difficult to extract distinguishing features. Studies [58,59] have improved the representation of the model for small defects by designing feature-enhancement modules and making use of multi-scale features. It has been shown to effectively resolve the abundant texture variations and small-sized defects on the target surface. Other studies [60,61] have improved the accuracy of defect detection by fusing feature maps of different levels and different sizes. The robustness of using multi-scale features is shown to adapt to the diversity of defect shapes. In further studies [62,63,64,65], researchers have obtained the full-scale features to detect defects of multiple scales using a multi-scale feature fusion network.

In defect detection, it is necessary to pay attention to the key areas of random defects, and it is also necessary to reduce background interference and illumination changes on defect detection. Researchers [66] have proposed an Adaptive Graph Channel Attention (AGCA) module to improve the feature representation ability. Study [67] uses Channel Attention Module (CAM) and Bidirectional Feature Fusion (BFFN) module to fully fuse features. It has shown that a combination of the two models can reduce the impact of complex environments. Study [68] uses the coordination attention (CA) module, which improves the ability of the network to locate defects.

From the above survey, it can be found that there is a gap in detecting the scale and location distributions of steel surface defects; defects with extreme scale distributions (such as tiny and giant defects) cannot be detected. In addition, devices with limited computing resources often have small storage capacity and computing power, and the design of some models often ignores the impact of model size and inference speed, making it difficult for models to run on hardware with limited computing power.

Therefore, to fully improve the lightweight and the detection ability of the model for defects of different scales, this study proposes a model DAssd-Net for steel surface defect detection.

## 3. Steel Surface Defect Detection Network DAssd-Net

We will introduce the overall framework of the proposed steel surface defect detection model based on dilated convolution and attention fusion modules. We will then introduce the model substructures, including the Dilated Convolution Aggregation Module (DCAM), Dilated Convolution and Channel Attention Fusion Module (DCM), and Dilated Convolution and Spatial Attention Fusion Module (DSM).

### 3.1. Overall Network Architecture

Designing a specific object detection model usually requires analyzing the distribution of sample boxes in the dataset. The center point coordinates of the sample boxes will be used to indicate the relative position of the defect examples in the image, while the width and height of the sample boxes will be used to indicate the size distribution of the defect examples. By analyzing the distribution of the center point and size of the detection boxes, a detection model tailored to the dataset is designed.

**Figure 1:**
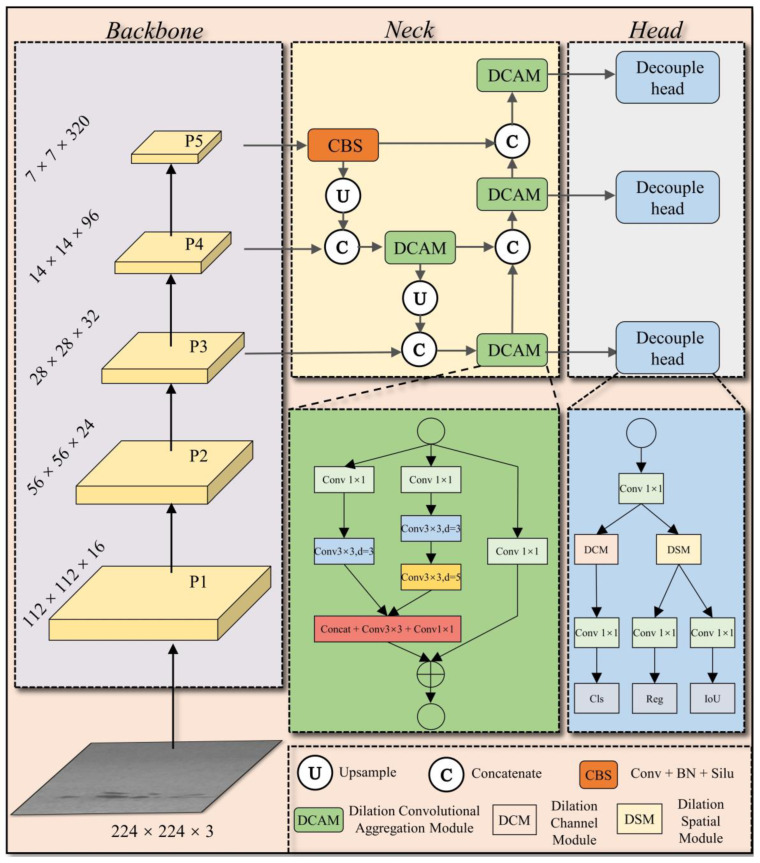
DAssd-Net structure of steel surface defect detection network.

The overall structure of the proposed steel surface defect detection network DAssd-Net is shown in Figure 1. We use the lightweight model MobileNetv2 as the backbone network for feature-extraction from steel surface defect images. To reduce the redundancy and increase the size of the receptive field of the network, the FPN structure composed of the DCAM module is proposed for feature fusion. To improve the network’s function for the important areas and channels of the image or feature map, we integrate the DSM and DCM modules in the detection head network. Such integration can identify the defect category and locations.

### 3.2. Dilated Convolution Aggregation Module (DCAM)

**Figure 2:**
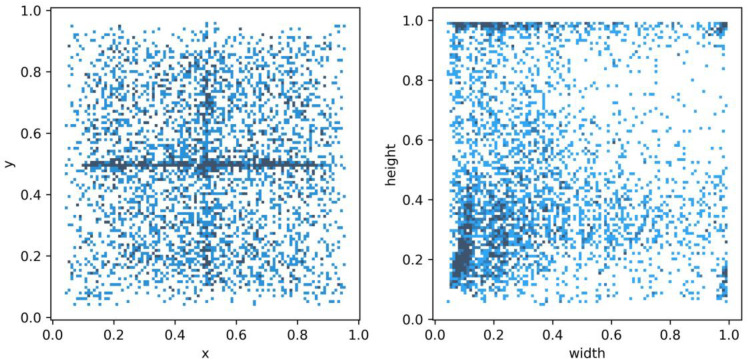
NEU-DET dataset labeled box x, y, width, height statistical information.

We count all the normalized label boxes x, y, width, and height information in the data set for statistics, and calculate the center point distribution and size distribution of all label boxes. In Figure 2, most of the center points of the label boxes are in the center of the y direction (y = 0.5), and the scale (i.e., height and width) distribution is mostly concentrated on small sizes, but there are also many large-scale label boxes. Due to the limited receptive field of ordinary convolution, it is difficult to detect large objects in the image. Dilated convolution is used to increase the receptive field without increasing the number of parameters. The expansion of the receptive field helps to detect large targets. When different dilation rates are selected, receptive fields of different sizes are obtained, along with multi-scale information.

**Figure 3:**
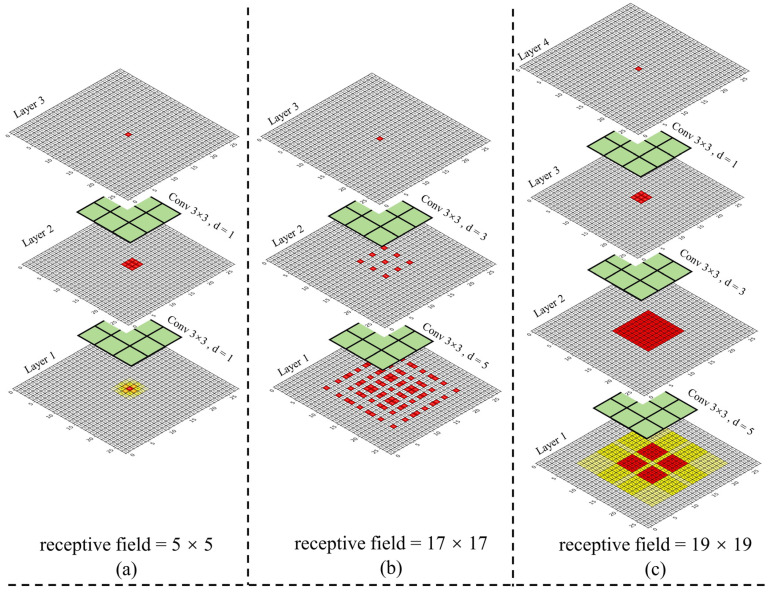
Receptive field analysis diagram when ordinary convolution and dilated convolution are stacked. The numbers in the figure are the number of times used by the convolution kernel. (**a**) the stacking result of two ordinary convolutions. (**b**) the dilated convolution stacking results with a dilation rate (3, 5). (**c**) the results of dilated convolution stacking with a dilation rate (1, 3, 5).

The size of the two-layer convolution kernel is 3 × 3, and the size of the receptive field after stacking the ordinary convolution with a step size of one is 5 × 5, as shown in Figure 3a. When the expansion rate is (3, 5), the size of the receptive field is 17 × 17. From Figure 3b, it can be found that there is a lack of correlation between the convolution results of this layer, resulting in the loss of local information and the gridding effect. To solve the problem, the Hybrid Dilated Convolution (HDC) [69] criterion is used to design the size of the expansion rate. We designed the expansion rate to be (1, 3, 5) and the perception size to be 19 × 19, as shown in Figure 3c, to avoid the gridding effect. In following equations, we assume that the convolution kernel size of the dilation convolution is k and the stride is one, then the size of the receptive field of the expansion convolution of the *i* + first layer is $rf_{i+1}$:

(1) $$k^{\prime }=k+(k-1)\times (d-1)$$

(2) $$rf_{i+1}=rf_{i}+(k^{\prime }-1)$$

where k is the size of the convolution kernel, $k^{\prime }$ is the size of the equivalent convolution kernel, d is the expansion rate, and rf is the size of the receptive field.

We have adopted a structure like the Receptive Field Block (RFB) [70], but due to the small size of the dataset image, we have redesigned a more streamlined Dilated Convolution Aggregation Module (DCAM). Specifically, we designed two dilated convolution branch structures, then concatenated the branch structures, and finally eliminated the gridding effect through a 3 × 3 convolution with an expansion rate of 1. This design has ensured the acquisition of local detailed information. The 1 × 1 convolution in DCAM is mainly to adjust the number of channels.

### 3.3. Dilated Convolution and Channel Attention Fusion Module (DCM)

**Figure 4:**
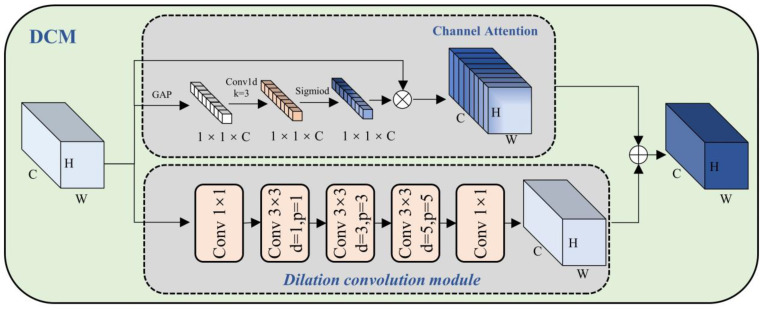
Schematic diagram of the structure of Dilated Convolution and Channel Attention Fusion Module (DCM).

In the CNN-based target detection model, to provide more information (such as color, texture, and shape) and better representation capabilities, a multi-channel (such as the number of channels C = 256) method is usually used. However, in the actual model, there may be a certain correlation between different channels, and there may be some redundant information. Channel attention is used to select important information and suppress redundant information [47,48]. As shown in Figure 4, we have designed the Dilated Convolution and Channel Attention Fusion Module (DCM), using the method of channel attention and dilated convolution fusion to improve the representation and generalization of the model.

A given feature map, $I_{c}\in {\mathbb{R}}^{H\times W\times C}$, will pass through two branches: the channel attention branch and dilation convolution branch. The channel attention branch will adaptively adjust the weight of each channel according to the importance of the channel to better capture the salient features in the input data. Use the channel-by-channel global average pooling operation to compress the channel dimension of $I_{c}$, and obtain the global information $z\in {\mathbb{R}}^{1\times 1\times C}$ between channels, and the c^th^ channel of z as follows:

(3) $$z_{c}=\frac{1}{H\times W}\sum_{i=i}^{H}\sum_{j=1}^{W}I_{c}^{}(i,j)$$

The one-dimensional convolution kernel is used to learn the dependencies between channels and normalize the weights through the sigmoid function. This operation produces the channel feature map $s\in {\mathbb{R}}^{1\times 1\times c}$:

(4) s=σ(Conv1d(z))

where Conv1d indicates a one-dimensional convolution kernel [71] with kernel size equal to three and padding equal to one. σ represents the sigmoid function.

Finally, the feature map $I_{c}$ is multiplied elementwise by the normalized channel weight s to obtain the output channel feature map $O_{c}\in {\mathbb{R}}^{H\times W\times C}$:

(5) $$O_{c}^{}=I_{c}^{}⊗s_{c}$$

where $I_{c}$ obtains $O_{d}\in {\mathbb{R}}^{H\times W\times C}$ after the dilation convolution operation with different expansion rates (1, 3, 5) of the stack. Adding $O_{c}$ and $O_{d}$ elementwise through the channel attention operation gives the fused feature map $O_{DCM}\in {\mathbb{R}}^{H\times W\times C}$:

(6) $$O_{DCM}=O_{d}⊕O_{c}$$

### 3.4. Dilated Convolution and Spatial Attention Fusion Module (DSM)

**Figure 5:**
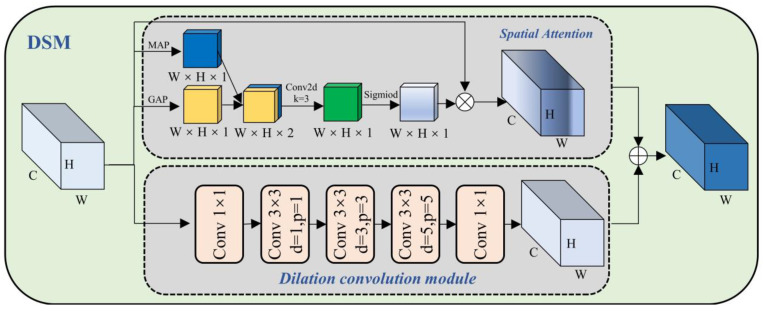
Schematic diagram of Dilated Convolution and Spatial Attention Fusion Module (DSM).

In steel surface defect detection, different types of defects often show different forms. For example, crazing defects often have a wavy texture shape, and inclusion defects often have irregular oval shapes. In addition to shapes, different types of defects have different sizes. Pitted-surface type defects are often large, while rolled-in scale type defects are often small. To better focus on the area of each different defect’s shape and size, as shown in Figure 5, we have proposed the Dilated Convolution and Spatial Attention Fusion Module (DSM). The module can better focus on the local area in the image, thereby improving the perception of local features, while increasing the receptive field.

A given feature map $I_{s}\in {\mathbb{R}}^{H\times W\times C}$ will go through the spatial attention branch and the Dilation convolution module branch. First, after a channel-based global average pooling and global max pooling, $g_{s}\in {\mathbb{R}}^{H\times W\times 1}$ and $m_{s}\in {\mathbb{R}}^{H\times W\times 1}$, are obtained, respectively:

(7) $$g_{s}=\frac{1}{C}\sum_{i=1}^{C}I_{s}(i)$$

(8) $$m_{s}=\max\left(I_{C}(1),I_{C}(2)\cdots I_{C}(H\times W)\right)$$

where $I_{C}(i)\in {\mathbb{R}}^{C},i=1\cdots H\times W$, $I_{C}(i),i=1\cdots H\times W$ represents the set of all channels in which each spatial pixel is located. $g_{s}$ is the average value in all channel sets where each spatial pixel is located. $m_{s}$ is the maximum value in all channel sets where each spatial pixel is located.

Then, $g_{s}$ and $m_{s}$ are spliced according to the channel direction to obtain $y_{s}\in {\mathbb{R}}^{H\times W\times 2}$. After $y_{s}$ is subjected to a 2D convolution operation with a kernel size equal to three, the dimension is reduced to one channel, and then the spatial attention feature map $c\in {\mathbb{R}}^{H\times W\times 1}$ is generated through the sigmoid function:

(9) $$y_{s}=Concat(g_{s},m_{s})$$

(10) c=σ(Conv2d(y))

where Concat represents the splicing operation by channel, Conv2d represents the 2D convolution operation, and σ represents the sigmoid function.

The feature map and the normalized spatial weight are multiplied elementwise to obtain the output channel feature map $O_{s}\in {\mathbb{R}}^{H\times W\times C}$:

(11) $$O_{s}=I_{s}⊗c_{s}$$

where $I_{s}$ is obtained by dilation convolution with different expansion rates (1, 3, 5) of the stack. Finally, $O_{d}\in {\mathbb{R}}^{H\times W\times C}$ is obtained by adding $O_{s}$ and $O_{d}$ after the spatial attention operation element by element to obtain the fusion of the subsequent feature map $O_{DSM}\in {\mathbb{R}}^{H\times W\times C}$:

(12) $$O_{DSM}=O_{d}⊕O_{s}$$

## 4. Experiment and Results Analysis

### 4.1. Experimental Configuration

#### 4.1.1. NEU-DET Dataset

This paper uses the steel surface defect detection public dataset NEU-DET [72], which includes a total of 1800 images of six types of defects, 300 images of each defect type, and the size of the image is 200 × 200 pixels. We have renamed the six defect types for convenience: C (crazing), RS (rolled-in scales), I (inclusion), P (patches), PS (pitted surface), and S (scratches).

**Figure 6:**
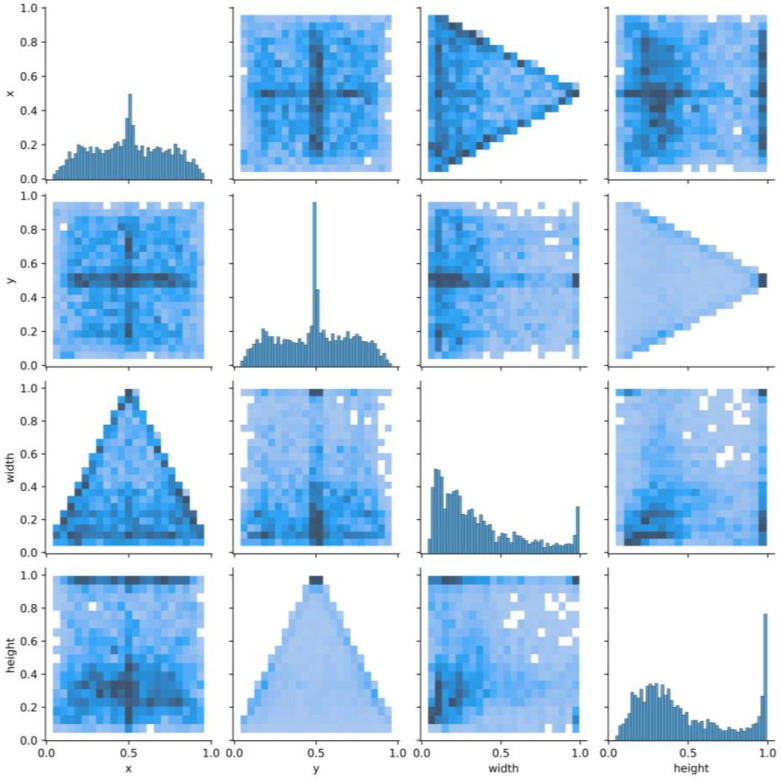
Correlation diagram of center point coordinates (x, y) and dimensions (width, height).

Through formula (13), the pixel value format $\left(x_{\min},y_{\min},x_{\max},y_{\max}\right)$ of the upper left corner and lower right corner of the data set label are converted into the center point and width and height format $\left(x_{c},y_{c},w,h\right)$.

(13) $$\begin{array}{c} x_{c}=[x_{\min}+(x_{\max}-x_{\min})/2]\times \frac{1}{width}\\ y_{c}=[y_{\min}+(y_{\max}-y_{\min})/2]\times \frac{1}{height}\\ w=(x_{\max}-x_{\min})\times \frac{1}{width}\\ h=(y_{\max}-y_{\min})\times \frac{1}{height} \end{array}$$

where width is the width of the image, and height is the height of the image. The pairwise relationship between the four attributes of x, y, width, and height is described. The diagonal line represents the histogram (distribution map) of each attribute, and the off-diagonal line represents the correlation between two different attributes. From Figure 6, it can be found that the center points of the target frame are mostly concentrated in the central area of the image. However, the width and height attributes are unevenly distributed, and there is a distribution at the maximum value, suggesting that there is a large-sized target box.

#### 4.1.2. Experimental Parameter Settings

The experiment is carried out on a PC with a 12th Gen Intel^®^ Core™ i5-12400F processor, NVIDIA GeForce RTX 3060 Ti GPU, CUDA 11.3, cuDNN 8.2.1, and Windows 10 operating system. The experimental code is written and debugged on Python Integrated Development Environment Pycharm, and the deep learning framework used is Pytorch. The number of training data sets is 1440, the number of verification data sets is 180, and the number of test data sets is 180. For the simulations, the initial learning rate is set to 0.01, the Adam optimizer is used, the momentum is set to 0.937, the cosine function learning rate decay method is used, and the total epoch is 300 rounds. The IOU loss uses the GIOU loss function [73], and the confidence loss and classification loss use the binary cross-entropy loss function.

### 4.2. Evaluation Criteria

The experiment mainly evaluates the model for accuracy and lightweight. AP (Average Precision) and mAP (mean Average Precision) are indicators used to evaluate the detection performance of a single category and the performance of the entire object detection system, respectively. Additionally, mAP is used as the final evaluation metric of model performance. We have analyzed the AP of each defect, mAP, model parameter size, theoretical amount of floating-point arithmetic (FLOPs), theoretical amount of multiply adds (MAdd), memory usage, and model storage size. For each category, all test images are sorted by confidence, and then each detection box is regarded as a positive example according to the confidence level from high to low, and it is matched with the ground truth box according to the IOU value. In the experiment, we set the IOU value to 0.5. If the IOU value is greater than a certain threshold, the detection box is regarded as a true case (TP), otherwise, it is regarded as a false positive case (FP). To calculate the accuracy and recall rate, it is necessary to judge whether the detection result is correct according to the IOU value of the detection frame and the real frame. We can calculate the AP value and mAP value of each category using the following formula:

(14) $$\begin{array}{c} precision=\frac{TP}{TP+FP}\\ recall=\frac{TP}{TP+FN}\\ AP=\int_{0}^{1}p(r)dr\\ mAP=\frac{1}{N}\sum_{i=1}^{N}AP_{i} \end{array}$$

where TP (True Positive) is the number of true cases, that is, the number of correct targets detected; FP (False Positive) is the number of false positive cases, that is, the number of false targets detected; FN (False Negative) is the number of false negative cases, that is, the number of false targets detected. Precision refers to the ratio of the number of detected positive samples to the number of all detected samples, and recall refers to the ratio of the number of detected positive samples to the number of all positive samples. p(r) is the precision value when the recall rate is *r*. *N* is the number of categories in the dataset.

### 4.3. Ablation Experiment

#### 4.3.1. Data Augmentation Strategies

We have used a detection network that does not use the data enhancement strategy as the baseline and considered the impact of the data enhancement strategy Mixup [74] and Mosaic [28] on the accuracy of model detection. In Table 1, we can see that the Mixup strategy does not bring about an improvement in accuracy, and the use of the mosaic strategy will bring about a 3.56% increase in mAP. The Mixup strategy randomly selects two images from each batch and mixes them in a certain ratio to generate a new image. This strategy generates a virtual blend of images, introducing the noise. The Mosaic data enhancement stitches four different images to form a new sample, thereby improving the diversity and representativeness of the data and helping to improve the generalization ability of the model. This paper selects to use the mosaic strategy as the data augmentation strategy for the experiments.

#### 4.3.2. DCM and DSM

We have evaluated the performance of DCM, DSM, and convolution on different detection head tasks. The neck network currently is a DCAM module. The proposed DCM and DSM can fuse force channel and spatial attention, aiming to have different attention regions for different detection tasks. In Table 2, when the classification task branch uses DSM and the regression task branch uses DCM, the highest mAP is achieved on the NEU-DET dataset. The DCM pays more attention to defects in crazing and rolled-in scales categories, while the DSM pays more attention to defects in inclusions and patches categories. This result shows that channel attention can capture features such as texture in the image, while spatial attention can capture features such as target positions and edges in the image.

**Figure 7:**
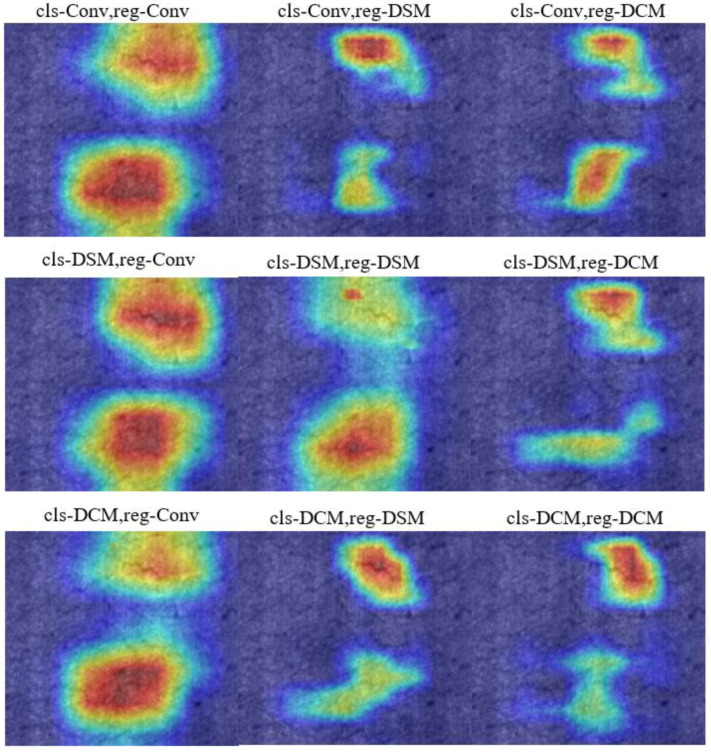
Comparison of heat map experiment results after fusion of DCM and DSM on different detection head tasks.

The heat map can visualize the prediction results of the model for each pixel, usually by means of color coding to represent the confidence of different pixels. Pixels with higher confidence are represented by warmer tones, such as red, and pixels with lower confidence are represented by cooler tones, such as blue. In addition, the heat map can also help us analyze the detection results of the model, such as judging which areas are easy to detect and which areas are easy to ignore. Figure 7 shows the heatmaps that visualize the fusion of DCM and DSM modules across different detection head tasks. When DSM is added to the regression head, the model can accurately locate the defect position. When the classification head adds the DCM module, the model can remove the redundant information of concern. In addition, using the DSM module can pay more attention to the spatial position of the target, and the DCM module can better remove redundant information. Through the visual analysis of the heat map of the model, it can be shown that DCM and DSM have the functions of accurately locating defect positions and removing redundant information.

**Figure 8:**
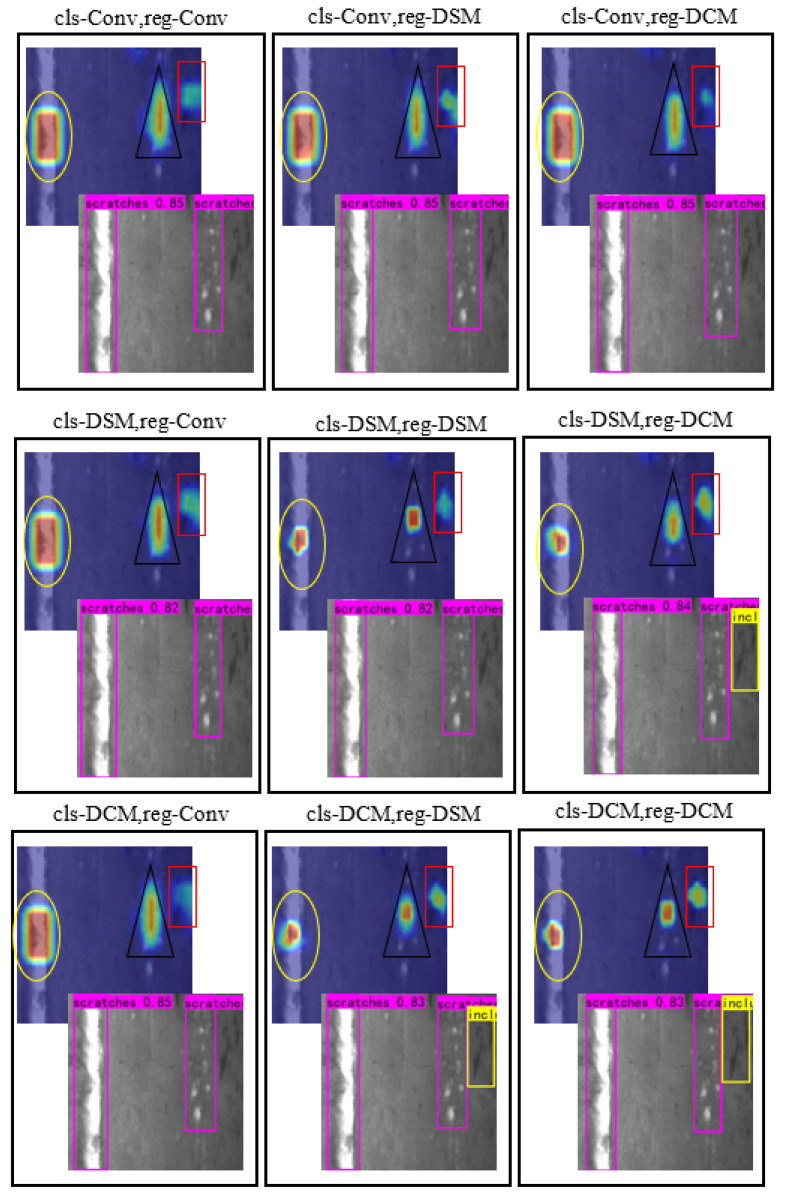
Comparison of experimental results of heat map details after fusion of DCM and DSM on different detection head tasks.

We have also performed heat map visualization analysis on images with both scratches and inclusion defects (Figure 8). The yellow ellipse in the image represents the region of interest of the large object heatmap, the black triangle represents the region of interest of the defect feature fuzzy object heatmap, and the red rectangle represents the region of interest of the heatmap containing the category. From the detection results, we can find that when one of the detection heads uses a common convolution structure, the model does not detect tiny defects. When DSM and DCM are used in combination, the model can more accurately identify the defect location and remove the redundant information of the defect. When the classification detection head uses DSM and the regression detection head uses DCM, the model pays more attention to small defects, while the degree of attention to other defects is more accurate and effective.

#### 4.3.3. Effect of Different Modules

To evaluate the impact of the specific modules of our proposed DAssd-Net model structure on detection accuracy, we have conducted experiments on different modules separately. In Table 3, when the DCAM module instead of CSPLayer is used in the feature fusion structure, the accuracy is increased by 2.58 mAP. It shows that the DCAM module can effectively increase the receptive field of the model and help the model better capture the context information of the target, thereby improving the detection performance of the model.

**Figure 9:**
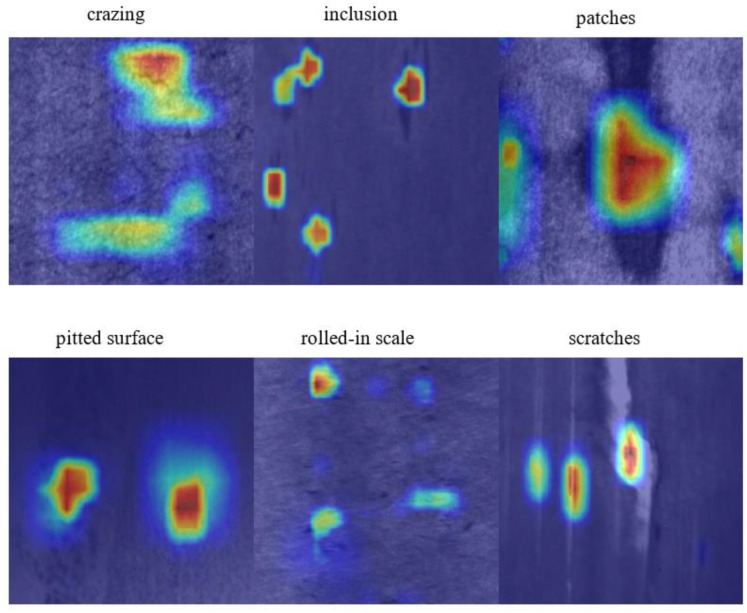
Heat map experiment results when DSM acts on the classification task branch and DCM acts on the regression task branch.

The integration of DSM and DCM modules in the model detection head can better capture features and improve model accuracy. We present the heatmap visualization results in Figure 9. In terms of model size, the DAssd-Net model structure replaces the ordinary convolution structure with a complex structure and many parameters, but with a significantly reduced model size.

### 4.4. Comparison with Other Models

**Figure 10:**
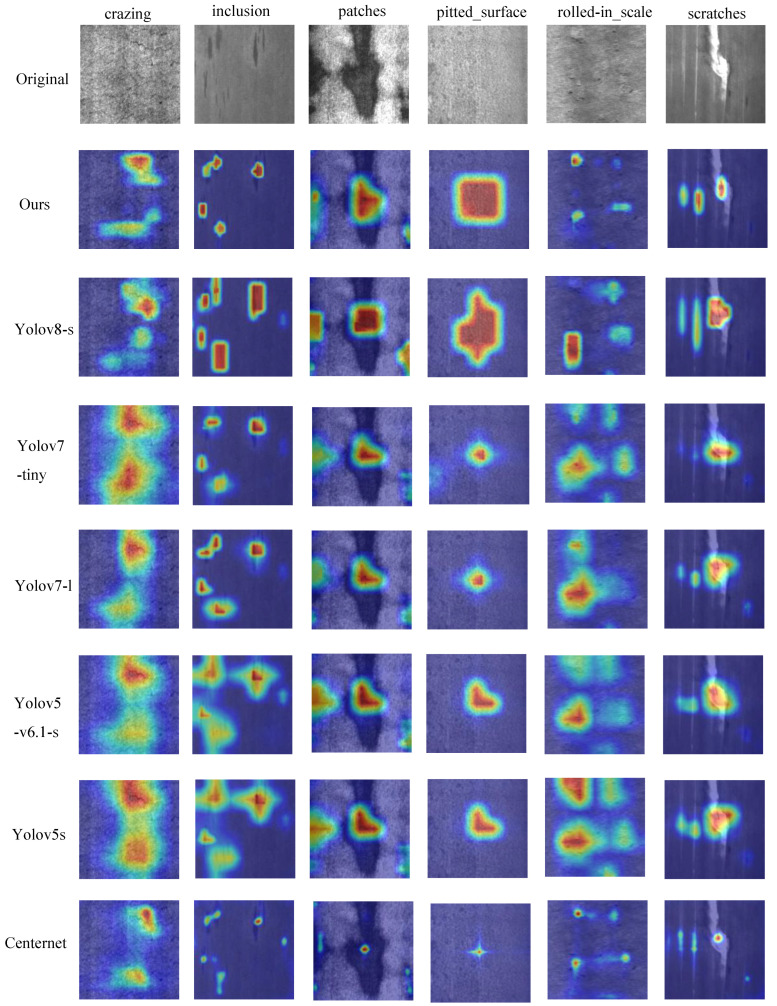
Comparison of heat map experimental results between the proposed model and the mainstream target detection model.

We have compared the proposed DAssd-Net model with other mainstream object detection models. These models include CenterNet [75], YOLOv5 [76], YOLOv5-v6.1 [77], YOLOv7 [29], and YOLOv8. The experiments are conducted using the same equipment and training strategy to compare the performance of steel surface defect detection in terms of accuracy and lightweight on the NEU-DET dataset. The heat map visualization results of different models are shown in Figure 10. Our proposed model can accurately identify the location of the target area with almost no redundant attention information. Other models do not pay enough attention to the center of the object.

Table 4 compares the accuracy of AP and mAP of different types of defects among different models. We can see that the DAssd-Net model we proposed can achieve the highest mAP. Except for the rolled-in scales defect category, our proposed models can achieve the highest accuracy. Compared with the newly proposed YOLOv8 model, our model has a 4.69% accuracy improvement.

Table 5 compares the different models for parameter quantity, FLOPs, Madd, Memory usage, and model storage size. It shows that the DAssd-Net model we proposed is superior to other models in terms of parameter quantity and model size and can realize the lightweight of the model. In terms of model complexity, the DAssd-Net model is superior to other models for a lower computational complexity and a higher operating efficiency.

**Figure 11:**
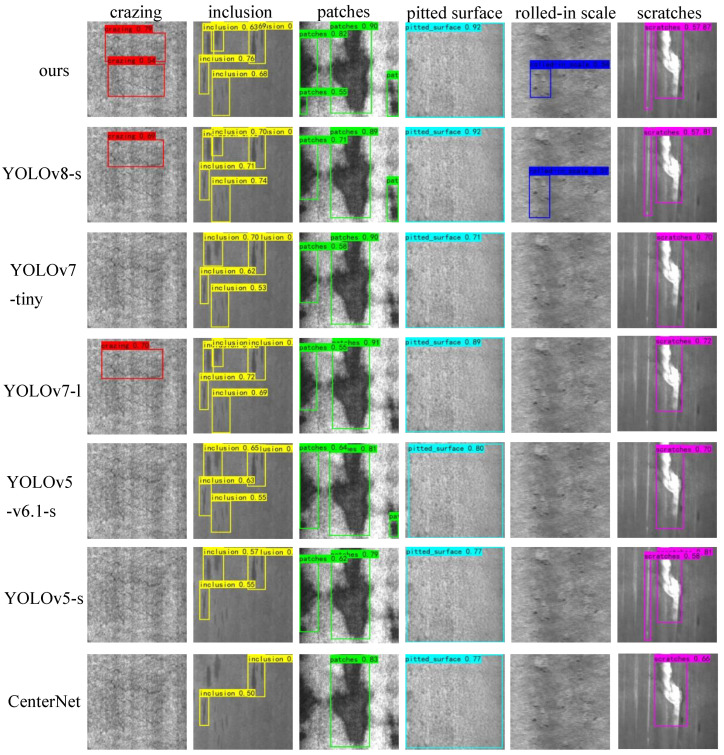
Comparison of the detection results between the proposed model and the mainstream target detection model.

The final detection results are shown in Figure 11. We compare our proposed model with current mainstream object detection models. We can find that the two defects of crazing and rolled-in scale are not detected in most target detection models. The YOLOv8 model and YOLOv7 model only detect one target of the crazing defect category, while our proposed model can detect two defect locations. In small target defects, other models have missed detection, such as inclusion and patched defect categories, while our model can detect small target defects. The results show our proposed model can accurately identify the defect locations.

## 5. Conclusions and Prospects

### 5.1. Conclusions

A lightweight steel surface defect detection model—DAssd-Net—is proposed. The model uses a multi-branch Dilated Convolution Aggregation Module (DCAM), which can effectively expand the receptive field and enhance contextual information fusion. Through experiments and heat map analysis, it can be found that:

- In the ablation experiments where DCM and DSM act on different detection head tasks, the model achieves the highest accuracy of 81.97% when DSM is fused with the classification detection head and when DCM is fused with the regression detection head. The heat map shows that the current model pays more attention to the spatial position of the target, is more sensitive to the detection of large targets, and suppresses the generation of redundant channel information.
- Compared with other mainstream target detection models, the DAssd-Net we proposed can achieve 81.97% mAP accuracy on the NEU-DET dataset, while the model size is only 18.7 MB. It is 4.69% higher than the latest YOLOv8 model mAP index, and the model size is 23.9 MB less, with advantages and lightweight.

### 5.2. Prospects

- The expansion rate of the dilated convolution determines the size of the receptive field of the convolution kernel, but different expansion rates correspond to different receptive field sizes, and the expansion rate needs to be manually adjusted to obtain the optimal receptive field size. In future work, we will study an expansion rate structure that can be adaptively designed according to the data set to improve the accurate acquisition of the size of the receptive field.
- The proposed model has good performance indicators on the PC side, but it needs to consider the model deployment to limited computing resources in the actual production process. In future research, it is necessary to use techniques such as model compression, model quantification, and knowledge distillation to deploy the model to meet the requirements of real-time and reliable steel surface defect detection.

## Figures and Tables

**Table 1:** Detection accuracy using different data augmentation strategies.

Baseline	Mixup	Mosaic	C	RS	I	P	PS	S	mAP
√			0.31	0.58	0.84	0.86	0.92	0.95	74.30%
√	√		0.33	0.53	0.83	0.87	0.94	0.94	74.13%
√		√	0.32	0.73	0.86	0.87	0.94	0.95	77.86%
√	√	√	0.35	0.65	0.81	0.91	0.95	0.96	77.21%

**Table 2:** The detection accuracy after the fusion of DCM and DSM on different detection head tasks.

Cls			Reg			AP						mAP
DCM	DSM	Conv	DCM	DSM	Conv	C	RS	I	P	PS	S	
√			√			0.38	0.70	0.82	0.89	0.94	0.98	78.24%
√				√		0.37	0.68	0.86	0.88	0.94	0.96	78.22%
√					√	0.39	0.73	0.86	0.89	0.96	0.97	79.72%
	√		√			0.54	0.70	0.86	0.92	0.95	0.95	81.97%
	√			√		0.41	0.69	0.88	0.93	0.94	0.97	80.33%
	√				√	0.35	0.70	0.83	0.88	0.94	0.97	77.81%
		√	√			0.40	0.72	0.85	0.90	0.92	0.94	78.84%
		√		√		0.40	0.71	0.87	0.93	0.94	0.96	80.28%
		√			√	0.46	0.72	0.85	0.92	0.93	0.95	80.44%

**Table 3:** Detection accuracy and model size after fusing different modules.

Baseline	DCAM	Cls-DSM	Reg-DCM	C	RS	I	P	PS	S	mAP	ModelSize (MB)
√				0.32	0.73	0.86	0.87	0.94	0.95	77.86%	46.0
√	√			0.46	0.72	0.85	0.92	0.93	0.95	80.44%	42.9
√	√	√		0.35	0.70	0.83	0.88	0.94	0.97	77.81%	30.8
√	√		√	0.40	0.72	0.85	0.90	0.92	0.94	78.84%	30.8
√	√	√	√	0.54	0.70	0.86	0.92	0.95	0.95	81.97%	18.7

**Table 4:** The comparison of the detection accuracy between the proposed model and the mainstream target detection model.

Model	C	RS	I	P	PS	S	mAP
CenterNet	0.36	0.67	0.81	0.88	0.91	0.95	76.34%
YOLOv5-s	0.30	0.72	0.79	0.87	0.90	0.92	75.02%
YOLOv5-v6.1-s	0.38	0.65	0.82	0.85	0.91	0.92	75.54%
YOLOv7-l	0.41	0.68	0.86	0.89	0.92	0.93	78.15%
YOLOv7-tiny	0.42	0.69	0.77	0.86	0.86	0.95	75.64%
YOLOv8-s	0.43	0.60	0.84	0.91	0.92	0.93	77.28%
ours	0.54	0.70	0.86	0.92	0.95	0.95	81.97%

**Table 5:** Performance comparison between the proposed model and the mainstream target detection model.

Model	Params (M)	Memory (MB)	Madd (G)	Flops (G)	MemR + W (MB)	ModelSize (MB)
CenterNet	32.67	116.85	9.69	4.24	323.72	124.0
YOLOv5-s	7.08	34.97	2.01	1.01	78.47	27.1
YOLOv5-v6.1-s	7.04	33.72	1.94	0.95	76.59	27.0
YOLOv7-l	37.22	86.40	12.84	6.42	328.74	142.0
YOLOv7-tiny	6.03	19.41	1.61	0.79	67.17	23.1
YOLOv8-s	11.14	25.58	3.49	1.75	97.15	42.6
ours	3.10	90.41	1.21	0.60	193.82	18.7

## Data Availability

Not applicable.

## References

[1] Zhang P., Wang J., Zhang F., Xu P., Li L., Li B. (2022). Design and Analysis of Welding Inspection Robot. Sci. Rep..

[2] Luo Q., Fang X., Liu L., Yang C., Sun Y. (2020). Automated Visual Defect Detection for Flat Steel Surface: A Survey. IEEE Trans. Instrum. Meas..

[3] Zhang Z., Ma P., Fang Y., Yang Z., Zhang N., Prashanth K.G., Jia Y. (2023). Effect of NiCoFeAlTi High Entropy Intermetallic Reinforcement Particle Size on the Microstructure and Mechanical Properties of CoCrFeMnNi High-Entropy Alloy Composites Fabricated by Selective Laser Melting. J. Alloys Compd..

[4] Ji J.Y., Zhang Z., Chen J., Zhang H., Zhang Y.Z., Lu H. (2023). Effect of Refractory Elements M (=Re, W, Mo or Ta) on the Diffusion Properties of Boron in Nickel-Based Single Crystal Superalloys. Vacuum.

[5] Liang M., Qin Y., Zhang D., Zhao F. (2022). Microstructural Evolution and Mechanical Properties of Vacuum Brazed TC4 Titanium Alloy Joints with Ti-Zr-Ni Filler Metal. J. Mater. Eng. Perform..

[6] Ma S., Li B., Ma Y., Zhang P., Xu P. (2022). Effect of Brazing Filler Metals and Welding Parameters on Laser Welding-Brazing Joints of WC-Co to S1045. Metals.

[7] Zhao F., Qin Y., Zhang D., Liang M. (2022). Effect of Filler Wire on Laser Lap Welding of Al-Si Coated 22MnB5 Hot Stamping Steel. J. Mater. Eng. Perform..

[8] Zhang Y., Chen J., Zhang W., Li C., Qiu C., Ding J., Lu H., Zhang K. (2023). Study of Spatter Net Forming Mechanism and Penetration Mode under Flexible Ring Mode Laser Welding. J. Mater. Res. Technol..

[9] Wang Z., Yang S., Peng Z., Gao Z. (2022). Effect of Defects in Laser Selective Melting of Ti-6Al-4V Alloy on Microstructure and Mechanical Properties after Heat Treatment. Opt. Laser Technol..

[10] Zhang T., Wang W., Ma Y., Fang N., Lin S., Li Z., Kou S. (2022). In Situ Observation of Microstructural and Inclusions Evolution in High-Strength Steel Deposited Metals with Various Rare Earth Pr Contents. Materials.

[11] Fan C., Yang S., Duan C., Zhu M., Bai Y. (2022). Microstructure and Mechanical Properties of 6061 Aluminum Alloy Laser-MIG Hybrid Welding Joint. J. Cent. South Univ..

[12] Zhang T., Yu H., Li Z., Kou S., Kim H.J., Tillmann W. (2021). Progress on Effects of Alloying Elements on Bainite Formation and Strength and Toughness of High Strength Steel Weld Metal. Mater. Res. Express.

[13] Zolfaghari A., Kolahan F. (2017). Reliability and Sensitivity of Visible Liquid Penetrant NDT for Inspection of Welded Components. Mater. Test..

[14] Chen Y., Kang Y., Feng B., Li Y., Cai X., Wang S. (2022). Automatic Defect Identification in Magnetic Particle Testing Using a Digital Model Aided De-Noising Method. Measurement.

[15] Cruz F.C., Simas Filho E.F., Albuquerque M.C.S., Silva I.C., Farias C.T.T., Gouvêa L.L. (2017). Efficient Feature Selection for Neural Network Based Detection of Flaws in Steel Welded Joints Using Ultrasound Testing. Ultrasonics.

[16] Canny J. (1986). A Computational Approach to Edge Detection. IEEE Trans. Pattern Anal. Mach. Intell..

[17] Bharati M.H., Liu J.J., MacGregor J.F. (2004). Image Texture Analysis: Methods and Comparisons. Chemom. Intell. Lab. Syst..

[18] Rosten E., Drummond T. Machine Learning for High-Speed Corner Detection. Proceedings of the 9th European Conference on Computer Vision (ECCV).

[19] Xu Y., Zhang D., Yang J.-Y. (2010). A Feature Extraction Method for Use with Bimodal Biometrics. Pattern Recognit..

[20] Cortes C., Vapnik V. (1995). Support-Vector Networks. Mach. Learn..

[21] Girshick R., Donahue J., Darrell T., Malik J. Rich Feature Hierarchies for Accurate Object Detection and Semantic Segmentation. Proceedings of the IEEE Conference on Computer Vision and Pattern Recognition (CVPR).

[22] Girshick R. Fast R-CNN. Proceedings of the IEEE International Conference on Computer Vision (ICCV).

[23] Ren S., He K., Girshick R., Sun J. (2017). Faster r-cnn: Towards real-time object detection with region proposal networks. IEEE T Pattern Anal..

[24] Liu W., Anguelov D., Erhan D., Szegedy C., Reed S., Fu C.-Y., Berg A.C. SSD: Single Shot MultiBox Detector. Proceedings of the 9th European Conference on Computer Vision (ECCV).

[25] Redmon J., Divvala S., Girshick R., Farhadi A. You Only Look Once: Unified, Real-Time Object Detection. Proceedings of the IEEE Conference on Computer Vision and Pattern Recognition (CVPR).

[26] Redmon J., Farhadi A. YOLO9000: Better, Faster, Stronger. Proceedings of the IEEE Conference on Computer Vision and Pattern Recognition (CVPR).

[27] Redmon J., Farhadi A. (2018). YOLOv3: An Incremental Improvement. arXiv.

[28] Bochkovskiy A., Wang C.-Y., Liao H.-Y.M. (2020). YOLOv4: Optimal Speed and Accuracy of Object Detection. arXiv.

[29] Wang C.-Y., Bochkovskiy A., Liao H.-Y.M. (2022). YOLOv7: Trainable Bag-of-Freebies Sets New State-of-the-Art for Re-al-Time Object Detectors. arXiv.

[30] Ge Z., Liu S., Wang F., Li Z., Sun J. (2021). YOLOX: Exceeding YOLO Series in 2021. arXiv.

[31] Li C., Li L., Jiang H., Weng K., Geng Y., Li L., Ke Z., Li Q., Cheng M., Nie W. (2022). YOLOv6: A Single-Stage Object Detection Framework for Industrial Applications. arXiv.

[32] Iandola F.N., Han S., Moskewicz M.W., Ashraf K., Dally W.J., Keutzer K. (2016). SqueezeNet: AlexNet-Level Accuracy with 50x Fewer Parameters and <0.5MB Model Size. arXiv.

[33] Howard A.G., Zhu M., Chen B., Kalenichenko D., Wang W., Weyand T., Andreetto M., Adam H. (2017). MobileNets: Efficient Convolutional Neural Networks for Mobile Vision Applications. arXiv.

[34] Sandler M., Howard A., Zhu M., Zhmoginov A., Chen L.-C. MobileNetV2: Inverted Residuals and Linear Bottle-necks. Proceedings of the IEEE Conference on Computer Vision and Pattern Recognition (CVPR).

[35] Howard A., Sandler M., Chu G., Chen L.-C., Chen B., Tan M., Wang W., Zhu Y., Pang R., Vasudevan V. Searching for MobileNetV3. Proceedings of the IEEE/CVF International Conference on Computer Vision (ICCV).

[36] Zhang X., Zhou X., Lin M., Sun J. ShuffleNet: An Extremely Efficient Convolutional Neural Network for Mobile Devices. Proceedings of the IEEE Conference on Computer Vision and Pattern Recognition (CVPR).

[37] Ma N., Zhang X., Zheng H.-T., Sun J. ShuffleNet V2: Practical Guidelines for Efficient CNN Architecture Design. Proceedings of the 9th European Conference on Computer Vision (ECCV).

[38] Tan M., Le Q. EfficientNet: Rethinking Model Scaling for Convolutional Neural Networks. Proceedings of the 36th International Conference on Machine Learning (PMLR).

[39] Tan M., Le Q. EfficientNetV2: Smaller Models and Faster Training. Proceedings of the 38th International Conference on Machine Learning (PMLR).

[40] Han K., Wang Y., Tian Q., Guo J., Xu C., Xu C. GhostNet: More Features From Cheap Operations. Proceedings of the IEEE Conference on Computer Vision and Pattern Recognition (CVPR).

[41] Tan M., Chen B., Pang R., Vasudevan V., Sandler M., Howard A., Le Q.V. MnasNet: Platform-Aware Neural Architecture Search for Mobile. Proceedings of the IEEE Conference on Computer Vision and Pattern Recognition (CVPR).

[42] Wang R.J., Li X., Ling C.X. (2018). Pelee: A Real-Time Object Detection System on Mobile Devices. arXiv.

[43] Lin T.Y., Dollar P., Girshick R., He K., Hariharan B., Belongie S. Feature Pyramid Networks for Object Detection. Proceedings of the IEEE Conference on Computer Vision and Pattern Recognition (CVPR).

[44] Liu S., Qi L., Qin H., Shi J., Jia J. Path Aggregation Network for Instance Segmentation. Proceedings of the IEEE Conference on Computer Vision and Pattern Recognition (CVPR).

[45] Tan M., Pang R., Le Q.V. EfficientDet: Scalable and Efficient Object Detection. Proceedings of the IEEE Conference on Computer Vision and Pattern Recognition (CVPR).

[46] Woo S., Park J., Lee J.Y., Kweon I.S. CBAM: Convolutional Block Attention Module. Proceedings of the 9th European Conference on Computer Vision (ECCV).

[47] Hu J., Shen L., Sun G. Squeeze-and-Excitation Networks. Proceedings of the IEEE Conference on Computer Vision and Pattern Recognition (CVPR).

[48] Wang Q., Wu B., Zhu P., Li P., Zuo W., Hu Q. ECA-Net: Efficient Channel Attention for Deep Convolutional Neural Networks. Proceedings of the IEEE Conference on Computer Vision and Pattern Recognition (CVPR).

[49] Li X., Wang W., Hu X., Yang J. Selective Kernel Networks. Proceedings of the IEEE Conference on Computer Vision and Pattern Recognition (CVPR).

[50] Yu F., Koltun V. (2015). Multi-Scale Context Aggregation by Dilated Convolutions. arXiv.

[51] Shi T., Kong J., Wang X., Liu Z., Zheng G. (2016). Improved Sobel Algorithm for Defect Detection of Rail Surfaces with Enhanced Efficiency and Accuracy. J. Cent. South Univ..

[52] Sharifzadeh M., Amirfattahi R., Sadri S., Alirezaee S., Ahmadi M. Detection of Steel Defect Using the Image Processing Algorithms. Proceedings of the 6th International Conference on Electrical Engineering (ICEENG).

[53] Win M., Bushroa A.R., Hassan M.A., Hilman N.M., Ide-Ektessabi A. (2015). A Contrast Adjustment Thresholding Method for Surface Defect Detection Based on Mesoscopy. IEEE. Trans. Ind. Inform..

[54] Liang Y., Xu K., Zhou P. (2020). Mask Gradient Response-Based Threshold Segmentation for Surface Defect Detection of Milled Aluminum Ingot. Sensors.

[55] Wu X., Xu K., Xu J. Application of Undecimated Wavelet Transform to Surface Defect Detection of Hot Rolled Steel Plates. Proceedings of the 2008 Congress on Image and Signal Processing.

[56] Saberironaghi A., Ren J., El-Gindy M. (2023). Defect Detection Methods for Industrial Products Using Deep Learning Techniques: A Review. Algorithms.

[57] Elhanashi A., Lowe D., Saponara S., Moshfeghi Y. Deep Learning Techniques to Identify and Classify COVID-19 Abnormalities on Chest X-ray Images. Proceedings of the Real-Time Image Processing and Deep Learning.

[58] Cui L., Jiang X., Xu M., Li W., Lv P., Zhou B. (2021). SDDNet: A Fast and Accurate Network for Surface Defect Detection. IEEE Trans. Instrum. Meas..

[59] Yu X., Lyu W., Zhou D., Wang C., Xu W. (2022). ES-Net: Efficient Scale-Aware Network for Tiny Defect Detection. IEEE Trans. Instrum. Meas..

[60] Liu G., Ma Q. (2023). Strip Steel Surface Defect Detecting Method Combined with a Multi-Layer Attention Mechanism Network. Meas. Sci. Technol..

[61] Zhang Y., Wang W., Li Z., Shu S., Lang X., Zhang T., Dong J. (2023). Development of a Cross-Scale Weighted Feature Fusion Network for Hot-Rolled Steel Surface Defect Detection. Eng. Appl. Artif. Intell..

[62] Lu H., Fang M., Qiu Y., Xu W. (2022). An Anchor-Free Defect Detector for Complex Background Based on Pixelwise Adaptive Multiscale Feature Fusion. IEEE Trans. Instrum. Meas..

[63] Zhang D., Hao X., Wang D., Qin C., Zhao B., Liang L., Liu W. (2023). An Efficient Lightweight Convolutional Neural Network for Industrial Surface Defect Detection. Artif. Intell. Rev..

[64] Liu R., Huang M., Gao Z., Cao Z., Cao P. (2023). MSC-DNet: An Efficient Detector with Multi-Scale Context for Defect Detection on Strip Steel Surface. Measurement.

[65] Tang R., Liu Z., Song Y., Duan G., Tan J. (2023). Hierarchical Multi-Scale Network for Cross-Scale Visual Defect Detection. J. Intell. Manuf..

[66] Xiang X., Wang Z., Zhang J., Xia Y., Chen P., Wang B. (2023). AGCA: An Adaptive Graph Channel Attention Module for Steel Surface Defect Detection. IEEE Trans. Instrum. Meas..

[67] Yu J., Cheng X., Li Q. (2022). Surface Defect Detection of Steel Strips Based on Anchor-Free Network With Channel Attention and Bidirectional Feature Fusion. IEEE Trans. Instrum. Meas..

[68] Chen H., Du Y., Fu Y., Zhu J., Zeng H. (2023). DCAM-Net: A Rapid Detection Network for Strip Steel Surface Defects Based on Deformable Convolution and Attention Mechanism. IEEE Trans. Instrum. Meas..

[69] Chen L.C., Papandreou G., Schroff F., Adam H. (2017). Rethinking Atrous Convolution for Semantic Image Segmentation. arXiv.

[70] Liu S., Huang D., Wang Y. Receptive Field Block Net for Accurate and Fast Object Detection. Proceedings of the 9th European Conference on Computer Vision (ECCV).

[71] Zhang W., Li C., Peng G., Chen Y., Zhang Z. (2018). A deep convolutional neural network with new training methods for bearing fault diagnosis under noisy environment and different working load. Mech. Syst. Signal Process..

[72] Song K., Yan Y. (2013). A Noise Robust Method Based on Completed Local Binary Patterns for Hot-Rolled Steel Strip Surface Defects. Appl. Surf. Sci..

[73] Rezatofighi H., Tsoi N., Gwak J., Sadeghian A., Reid I., Savarese S. Generalized Intersection Over Union: A Metric and a Loss for Bounding Box Regression. Proceedings of the IEEE Conference on Computer Vision and Pattern Recognition (CVPR).

[74] Zhang H., Cisse M., Dauphin Y.N., Lopez-Paz D. (2017). Mixup: Beyond Empirical Risk Minimization. arXiv.

[75] Duan K., Bai S., Xie L., Qi H., Huang Q., Tian Q. CenterNet: Keypoint Triplets for Object Detection. Proceedings of the IEEE/CVF International Conference on Computer Vision (ICCV).

[76] YOLOv5 in PyTorch. https:/github.com/ultralytics/yolov5.

[77] YOLOv5-v6.1—TensorRT, TensorFlow Edge TPU and OpenVINO Export and Inference. https://github.com/ultralytics/yolov5/releases/tag/v6.1.

